# Ocean sound levels in the northeast Pacific recorded from an autonomous underwater glider

**DOI:** 10.1371/journal.pone.0225325

**Published:** 2019-11-20

**Authors:** Joseph H. Haxel, Haru Matsumoto, Christian Meinig, Gabriella Kalbach, T.-K. Lau, Robert P. Dziak, Scott Stalin

**Affiliations:** 1 Oregon State University and NOAA Pacific Marine Environmental Laboratory, Newport, Oregon, United States of America; 2 NOAA Pacific Marine Environmental Laboratory, Seattle, Washington, United States of America; 3 Department of Natural Sciences, California State University of Monterey Bay, Marina, California, United States of America; 4 NOAA Pacific Marine Environmental Laboratory, Newport, Oregon, United States of America; University of Waikato, NEW ZEALAND

## Abstract

Ocean gliders are a quiet and efficient mobile autonomous platform for passive acoustic monitoring and oceanographic measurements in remote marine environments. During July 20—August 6 2012, we used a Teledyne Webb Research Slocum G2 glider equipped with a hydrophone logging system to record ocean sound along a 458 km north to south traverse of the outer continental shelf break along the U.S. Pacific Northwest coast. Glider derived recordings yielded a unique perspective on the variation of ambient sound with depth, where natural wind generated surface processes were identified as a dominant acoustic contributor to spectral levels in the region. Near and far-field vessel radiated noise were also found to add significant energy to ambient conditions. Spatially distributed measurements of ambient sound levels recorded from the glider were consistent with long-term spectral estimates from fixed station, deep ocean hydrophone array measurements during the 1990–2000’s in the region. Ocean sound level measurements captured by a mobile glider are shown to be an effective and valuable asset for describing ocean surface wind conditions and characterizing spatial and temporal changes in the underwater acoustic environment over a broad regional scale.

## Introduction

Battery-powered, underwater gliders have emerged as an effective mobile technology platform for oceanographic data collection, covering vast areas of remote oceans with a stamina of months at a fraction of the cost of comparable measurements from ship based operations [1–4]. Using electric buoyancy engine technology, ocean gliders pump oil between an internal reservoir within the vehicle’s vacuum-sealed housing and an external bladder. The movement of oil in and out of the housing controls the density of the glider, thereby affecting the buoyancy force that powers its vertical motion in the water column. Wings convert hydrodynamic lift to a horizontal force that propels the glider forward, while rudder systems and mass balance controls enable horizontal navigation [2, 3]. The buoyancy driven descent/ascent, slow glide speeds (25 cm/s) and extended endurance capability to cover hundreds to several thousands of kilometers, make ocean gliders an attractive mobile platform for passive acoustic research when compared to louder, propulsion-driven autonomous underwater vehicles (AUVs) [5]. The glider’s stealth mobility and dive capabilities (up to 6000 m on Deepglider^™^) provide unique advantages for ecological studies of acoustically active animals by minimizing disturbance to sensitive marine mammals and fish that may otherwise avoid the noise emissions from louder vehicle platforms operating in remote and acoustically undisturbed ocean areas.

The use of ocean gliders as mobile platforms for bioacoustic recordings coupled with other in-situ environmental oceanographic sensors (e.g. temperature, salinity, optical backscatter) has provided important new ecological insights for behavioral studies of low frequency baleen whales [6–8], high frequency beaked whales [9], tracking of sperm whales and other odontocetes [10], and acoustically active fish [11] throughout a range of mesopelagic and coastal habitats. An ocean glider equipped with a hydrophone logging system provided valuable information for a geophysical study describing the acoustic signals radiating from eruptive processes at a remote submarine volcano in the western Pacific Ocean [12], and acoustic recordings from an ocean glider in the Mediterranean Sea have recently been shown to accurately predict surface wind speeds [13]. Yet, despite the onset use of underwater gliders for acoustics around 2006, little or no research has addressed measurements of ambient noise level conditions. This is likely owed to the extensive amount of self-noise produced by the gliders during operation, which can be largely ignored for marine mammal, fish and other signal identification research, but must be addressed in time series measurement and characterization of ambient sound levels. Furthermore, ocean sound has been identified as an Essential Ocean Variable (EOV) within the context of the Global Ocean Observing System (GOOS), where underwater gliders may provide a new, effective platform for sound level measurements across regional spatial scales.

Here we demonstrate a unique application for glider-based, passive acoustic recordings to measure spatial and short-term temporal patterns in underwater ambient sound levels along a broad region of the North American continental shelf break. Acoustic characterizations and sound level measurements in the northeast Pacific from fixed hydrophone stations have provided for robust time series descriptions of noise levels and long-term comparisons spanning decades [14–17]. The acoustic recordings presented in this study collected from a glider provide a spatial distribution or “snapshot” of ocean sound measurements at varying depths within a similar regional context to compare with fixed station sound level measurements in the northeast Pacific from 1994–2007 [18]. Additionally, the relative contributions from natural, wind related surface processes and anthropogenic noise radiated by vessels illuminate the frequency and time dependent importance of these dominant sound sources along the outer continental shelf of the Pacific Northwest USA.

## Data and methods

### Glider mission

**Fig 1:**
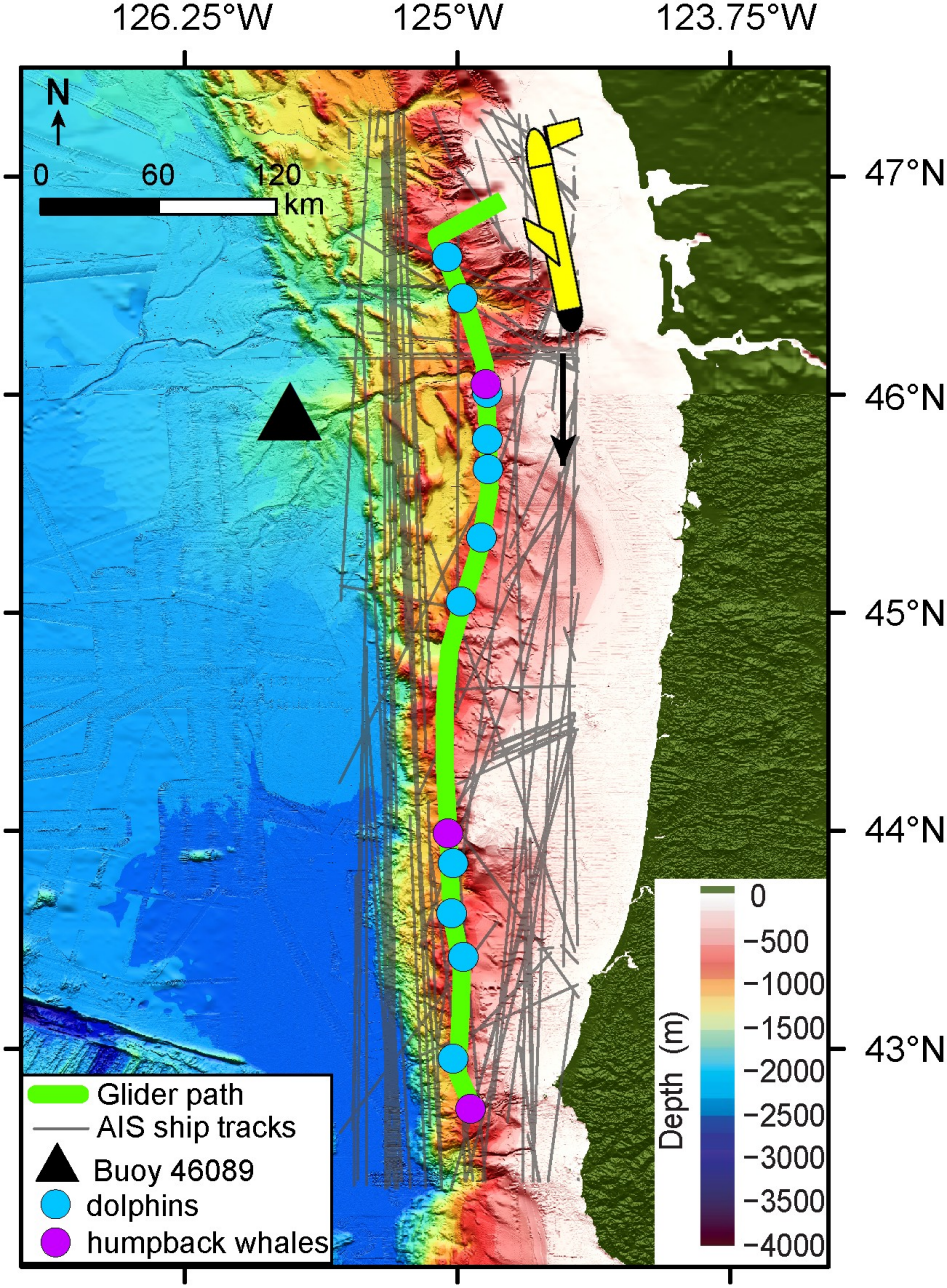
Map of the north-south glider mission down the Oregon coast. Green path shows the position of the glider. The black triangle marks the position of buoy 46089 used for wind speeds and AIS ship tracks are shown as grey lines surrounding the glider path. Circles indicate positions for vocal encounters where Pacific white-sided dolphins (blue) and humpback whales (magenta) where observed in the acoustic records. Bathymetry map was compiled and generated by Susan Merle (NOAA-PMEL/ Oregon State University) using openly accessible bathymetry data from the NOAA National Centers for Environmental Information (NCEI) and Marine Geoscience Data System (MGDS).

In 2012, the National Oceanic and Atmospheric Administration’s (NOAA) Pacific Marine Environmental Laboratory operated a 1000-m-rated, Teledyne Marine G2 Slocum ocean glider down the continental shelf break from Grays Harbor, Washington to Brookings, Oregon (Fig 1). The glider was flown entirely within U.S. exclusive economic zone (EEZ) waters, not requiring a permit for access, as part of a larger NOAA effort to characterize ocean acidification along the west coast of the United States. Since the main objective of the mission was for oceanographic measurements, and vocal encounters with marine mammals were passive and coincidental, animal care and use committee approval was also not required. In addition to a suite of environmental sensors for ocean acidification research, the glider was equipped with a hydrophone and acoustic data logging system during an 18-day mission (July 20^th^-August 6^th^) from north to south along the 1000 m depth contour of the outer continental shelf break. Over the course of this nearly three-week period, the glider performed a continuous series of 402 sawtooth dive profiles (i.e. “yo’s”) to a median dive depth of 625 m while covering a horizontal distance of 458 km at an average horizontal speed of 32 cm/s. The glider was not operated to its maximum depth rating of 1000 m to avoid collision with bathymetric complexity along the shelf break.

The glider was outfitted with a single, calibrated, omni-directional hydrophone (HTI92WB –175 dB *re* 1V/1μPa) fastened externally on the dorsal mid-section of the glider body using a semi-soft rubber mount. Previous work has shown that acoustic receiver performance from a hydrophone mounted on the dorsal mid-section of the Slocum glider is largely unaffected by the vehicle attitude and orientation [19], and therefore in a suitable position for ambient noise level measurements. The hydrophone sensor includes a built-in single pole high pass filter with corner frequency at 50 Hz. Prior to digitization, the acoustic signal was pre-whitened to reduce the ambient noise spectrum below 20 Hz so that the 16-bit dynamic range of the acquisition system can be fully utilized. The pre-amplifier consists of a series of gain stages with filters including two one-pole high-pass filters with cutoff frequencies at 1 Hz and 20 Hz, respectively. The last stage of the pre-amplifier is an 8-pole elliptical anti-aliasing filter with a cut-off frequency (*f*_*c*_) at 4400 Hz and a stop band rejection ratio of -82 dB at 1.5 x *f*_*c*_. The conditioned signal was then digitized by a data logging system at 10 kHz sample rate and 16-bit resolution. Data is recorded continuously with each acoustic data file 12 MB in size and archived every 10 minutes from buffer storage onto a 32 GB compact flashcard. At the apogee of every third dive in a sequence, the glider would rise to the sea surface to transmit summary data of oceanographic measurements (e.g. conductivity, temperature and depth (CTD), turbidity, chlorophyll), navigation data (e.g. global positioning system (GPS) location, heading) and glider system diagnostics (e.g. vacuum, battery power) back to shore via satellite telemetry. The acoustic system, with the exception of battery power, was installed independent of the glider scientific logging system and therefore does not share circuitry that could introduce noise from other instrument sampling. Due to the large size of the files, no acoustic data was transmitted in real time during glider surfacing.

**Fig 2:**
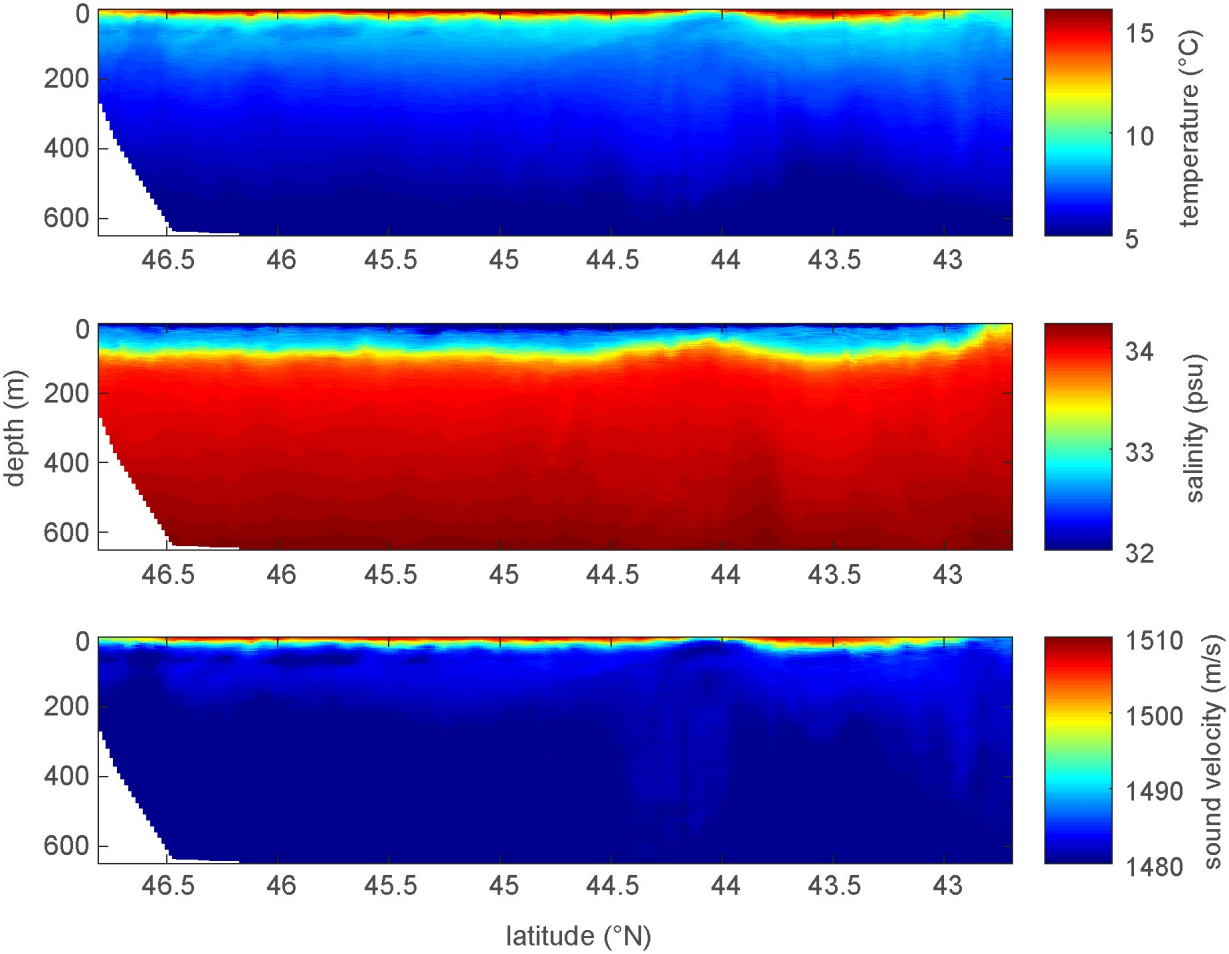
Two-dimensional cross sectional views of environmental data collected along the glider flight path. Water temperature (upper), salinity (mid), and calculated sound velocity (lower) measurements show the 2-D spatial structure of water column properties along the continental shelf break to 650 m depth.

The Glider CTD provided in-situ depth profile measurements of water column properties to 650 m during glider “yo’s” at 1 Hz sample rate. Interpolated 2-dimensional cross section views of temperature, salinity and calculated sound velocity along the entire glider flight path (Fig 2) indicate relatively stable environmental conditions with strong oceanographic stratification during most of the mission except during the period when the glider was located between 44.5°– 44° N where colder water temperatures and higher salinities were observed near the surface.

### Glider self-noise

Despite the quieter, buoyancy driven method of locomotion compared to propeller driven AUVs, significant mechanical system noise is intermittently generated by the glider as it navigates along its “sawtooth” three dimensional course [12]. These discrete glider sounds contaminate passive acoustic recordings, masking acoustic detections of target sources [10] and introduce significant bias to ambient noise level measurements if not adequately accounted for. Slocum glider self-noises are readily identified with distinct spectral characteristics [10, 12] and elevated amplitudes that were either filtered and smoothed (rudder noise) or annotated and removed from the passive acoustic time series as data loss (pump noise, pitch battery noise).

An electric servo-motor controls the glider’s rudder for directional adjustments, emitting distinct sounds at irregular intervals that are not logged by the glider’s navigation system. Rudder noise contamination in the acoustic record was characterized by a wide-band signal [10] with elevated harmonic energy in discrete frequency bands of *f*_*1r*_ < 1000 Hz and 2600 Hz < *f*_*2r*_ < 4400 Hz, that occurred in short (~0.25 sec) and long (~0.6 sec) durations. The longer duration rudder noise was observed most often at the top of dives within the first 100 m of the glider descent, resulting from sustained course corrections in the stronger near surface currents. Meanwhile, the shorter duration rudder noise was found more consistently throughout the dive profiles associated with minor course adjustments. To address rudder noise contamination, rudder-generated signals were identified and removed from the acoustic time series through an automated energy summation detection algorithm [20], and weighted smoothing function (S1 Appendix). Comparisons of spectral levels prior to the smoothing correction for rudder noise indicate a reduction in noise levels of up to 30 dB re 1 μPa^2^/Hz in particular frequency bands most influenced by the rudder sound.

Another problematic source of system noise contamination was generated by the buoyancy engine pumping oil to and from the external bladder reservoir and glider housing to initiate dives and ascents. The glider pump noise at the top and bottom of the dives consists of a long duration (10’s of seconds), high amplitude mechanical sound that is easily recognized [10] but unlike the short duration rudder noise cannot be removed due to its longevity, therefore resulting in acoustic data loss. Similar acoustic data loss periods result from electric servo motors shifting the center of mass of the glider pressure housing by moving battery packs to assist in dives or course turns and major corrections.

### Surface winds and vessel data

Wind surface measurements during the glider mission were collected from a NOAA surface buoy, National Data Buoy Center (NDBC) station 46089, 160 km WNW off the coast of Tillamook, Oregon (Fig 1). Buoy wind speeds were measured 5 m above sea level and averaged over an 8-minute period at the top of every hour. The buoy measurements were used in this study as a proxy for regional wind conditions affecting the area traversed by the glider. Although three other similar NOAA buoys were at times within closer range to the glider’s path, none of them were operational for surface wind measurements during the mission period. To assess the validity of buoy 46089 wind speeds as a proxy for regional conditions, satellite derived meridional (N-S) wind values, the strongest directional component of wind in this region during the summer upwelling season [21], were acquired from the microwave scatterometer ASCAT measuring global surface winds aboard the EUMETSATS Metop-A satellite (https://coastwatch.pfeg.noaa.gov/). ASCAT winds tracked the glider throughout its mission, providing more temporally coarse, but spatially consistent daily averages of local surface wind measurements on a 0.25° spatial scale.

Vessel traffic information surrounding the glider mission space was downloaded from NOAA and the Bureau of Ocean Energy Management’s (BOEM) MarineCadastre.gov automatic identification system (AIS) database (https://marinecadastre.gov/ais/). Raw AIS fixes including latitude, longitude, and time for a variety of vessels (e.g. container ships, tow, commercial fishing) were compiled for the glider area during the mission time frame and then transformed into daily tracklines (Fig 1) using the NOAA/BOEM AIS Track Builder 2.1.1 software in ArcGIS (https://marinecadastre.gov/ais/). A daily metric for localized vessel activity was calculated using the density of tracklines within a 50 km buffer of the daily median glider position. A 50 km buffer distance for local ship traffic was used to account for the coarse temporal and spatial resolution associated with a daily median values for the glider location and daily vessel tracks. This is based on a doubling of the average total distance traveled by the glider during a single day (27 km/day). Using a coarse transmission loss approximation TL = 15 log_10_(r), where TL is transmission loss and r is range from source to receiver, a 50 km daily buffer size accounts for 70 dB of transmission loss from vessel traffic sources to the glider at the most extreme ranges. This is a conservative estimate based on this simple approximation as spreading loss is likely much higher when considering propagation paths. Therefore, the loudest, low frequency ship noise radiated from large commercial vessels with source levels near 180 dB *re* 1 μPa [22, 23] would result in received levels within the range of sensitivity for the glider hydrophone at 110 dB *re* 1 μPa even at the most extreme ranges. The daily value of the total number of combined vessel-trackline kilometers within the 50 km buffer of the glider position was used for comparisons with acoustic conditions to determine the influence of localized ship traffic on ambient sound levels. Buffer ranges up to 80 km were explored without significant changes in results due to propagation energy loss effects.

## Analysis and results

### Sound level analysis and characterization

**Fig 3:**
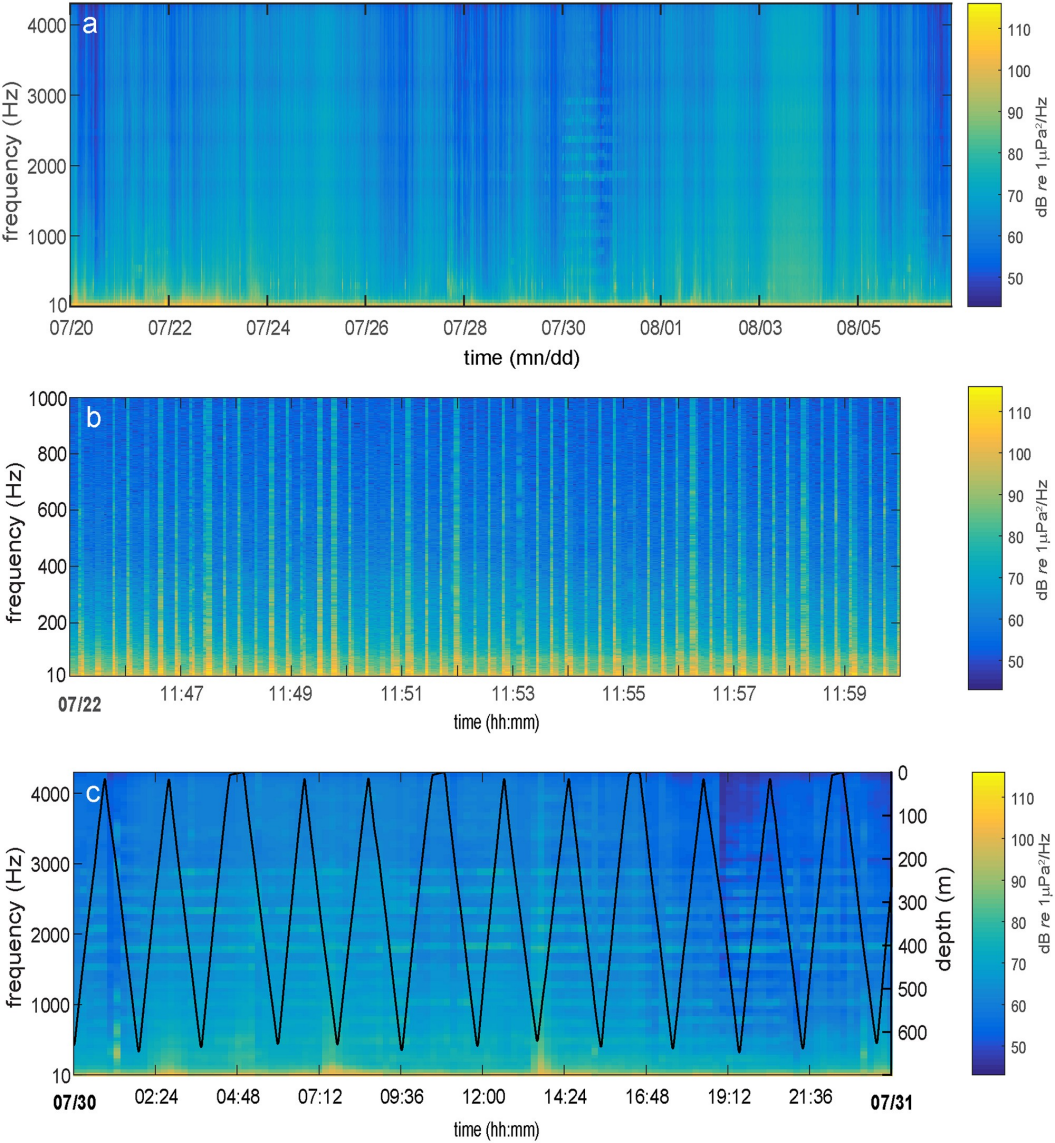
Glider spectrograms during the mission and glider dive profiles on July 30, 2012. **a)** A spectrogram calculated from 4 second hanning windows with no overlap showing the spectral energy content recorded during the 18 day glider mission duration. Broadband periods of elevated acoustic energy levels associated with higher wind speeds are observed over 1–3 day periods throughout the mission. **b)** A 15 minute period showing airgun explosion signals (10–1000 Hz) recorded by the glider near the beginning of the mission on July 22, 2012. **c)** A 1 day long spectrogram from July 30 showing the acoustic influence of a nearby vessel appearing as discrete bands in energy up to 3 kHz. The glider’s dive profiles throughout the day are also shown.

Acoustic data were converted to absolute pressure using a calibration correction for the hydrophone acquisition system response and sensitivity prior to analysis. Acoustic power spectral density (PSD) was calculated using a 4-second hanning window, no padding and no overlap resulting in PSD values that when combined with the average vehicle speed of 32 cm/s provide a spatial resolution on the order of 1.25 meter. Spectral levels from 10 Hz to the upper corner frequency roll-off *f*_*c*_ = 4400 Hz plotted over the duration of the glider mission (Fig 3a) show periods of episodic wideband noise level increases often lasting several days from wind generated surface processes (e.g. August 3–4). Acoustic emissions from nearby vessels were also readily observed, at times occurring as discrete harmonic banding lasting several hours (e.g. July 30). During the first three days of the glider’s mission (July 20-July 23 2012), an active seismic airgun study was underway on the research vessel *R/V Marcus G*. *Langseth* within the coastal shelf region of southwest Washington [24]. The glider hydrophone system recorded thousands of high amplitude, low frequency (*f*<100Hz) airgun explosions during this time (Fig 3b). When the glider was closest to the airgun survey region (July 20), many of the received airgun signals were of sufficient amplitude to saturate the sensor resulting in a clipped record that prohibits an absolute measurement of the received sound levels. Therefore, noise level estimates from July 20, 2012 under-represent true sound levels associated with the nearby seismic survey. By July 23, the *R/V Langseth* had completed their survey and the glider was located off the Columbia River (46.1°N) where it continued its southward progression recording at levels within the dynamic range of the logging system for the duration of the mission.

**Fig 4:**
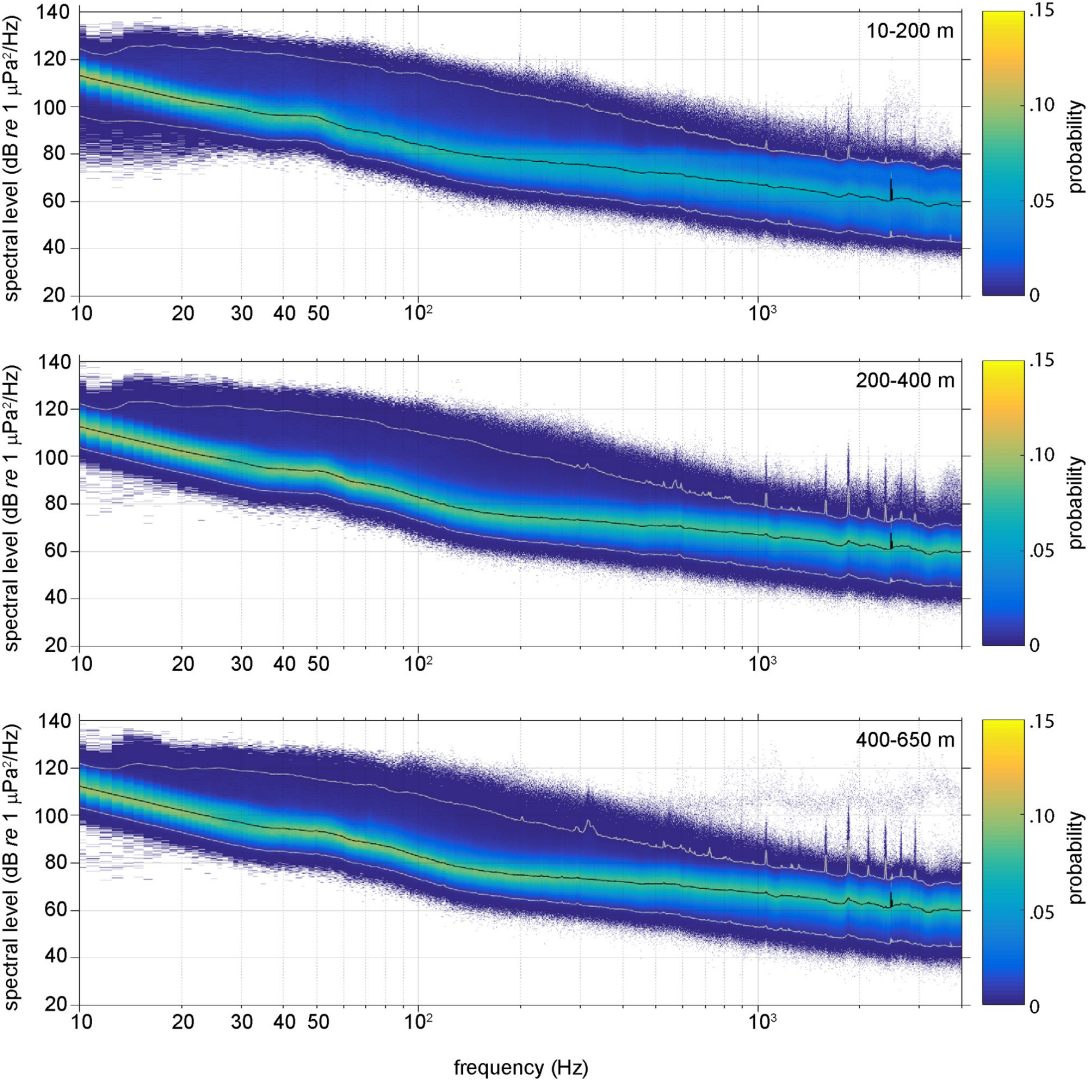
Spectral probability distributions (SPD) of acoustic energy in 3 depth intervals. SPDs calculated from 4 second hann windows, no overlap, as recorded by the glider at 3 distinct depth intervals; shallow (10–200 m); mid (200–400 m); and deep (400–650 m). Median (50^th^ percentile) values shown as black lines and 99^th^ and 1^st^ percentiles shown as white lines.

A detailed example of the acoustic effects of nearby ship-radiated noise is shown in Fig 3c. Harmonic banding associated with propeller cavitation was observed in frequencies up to 3 kHz, lasting throughout the day on July 30, 2012. The path of the glider dive profile revealed the strong persistence in amplitude and spectral structure of the vessel noise despite minimal variation in frequency associated with the hydrophone receiver depth and spatial relationship to the source. Empirical spectral probability densities from 4 second PSD windows (SPD; [25]) at 1 Hz resolution and binned according to 3 depth zones (10–200 m; 200–400 m; > 400 m) show the depth dependent variability in sound level distributions (Fig 4). The spectral slope of the median 50^th^ percentile curves representing “typical” acoustic conditions within the distributions remained consistent between the mid and deep water depths indicating there was little change in the frequency distribution of acoustic energy along the glider depth profiles below 200 m. Within the shallow depth interval (< 200 m), energy levels are less well constrained with lower levels of probability distributed more broadly around the median acoustic energy value at each frequency. In the shallow SPD for *f* < 30 Hz, some noise levels are observed at much lower amplitudes than the mid and deep water recordings. This is likely a flow noise effect as the glider speed is reduced near the surface. Additionally, the glider never reached the depths of the low velocity sound channel where more efficient propagation from distant low frequency sources could strongly affect received levels as a function of depth. Instead, spectral distributions show a characteristic broad, low frequency rise at all depths centered between 40 Hz < *f* < 60 Hz that we attribute to persistent regional scale commercial shipping activity [26, 27]. SPDs also reveal elevated spikes in acoustic energy levels in the 2–4 kHz frequency band (Fig 4) associated with an unknown, active low-mid frequency sound source in the glider area off the southern Oregon coast. On August 04, 2012 from 10:00–15:30, the glider recorded a series of discrete tonal sounds with received levels around 80–100 dB lasting 2 seconds and separated by 8 seconds that increased in frequency from roughly 200 Hz up to 4 kHz. The lower frequency tones from the sound source (< 1 kHz) are not resolved in the SPD plots due to the regular occurrence of noise levels at similar amplitudes.

Non-acoustic pressure fluctuations at the hydrophone sensor interface caused by turbulent eddy vortex shedding from the glider as it moves through the water results in flow-noise contamination in certain low frequencies of the acoustic recordings. The significant change to an upward slope in spectral levels below ~ 50 Hz (Fig 4) is attributed to persistent flow-noise caused by glider turbulence present during both ascent and descent phases of the dives. Relocating the hydrophone sensor in front of the glider-induced turbulent field at the leading edge of the vehicle will mitigate this type of flow-noise contamination in future missions. Bioacoustic signals, described in more detail in the following section, were observed infrequently throughout the data set with none of these sounds recurring or of high enough amplitude to significantly affect spectral noise level distributions.

### Environmental, anthropogenic and biological sources

**Fig 5:**
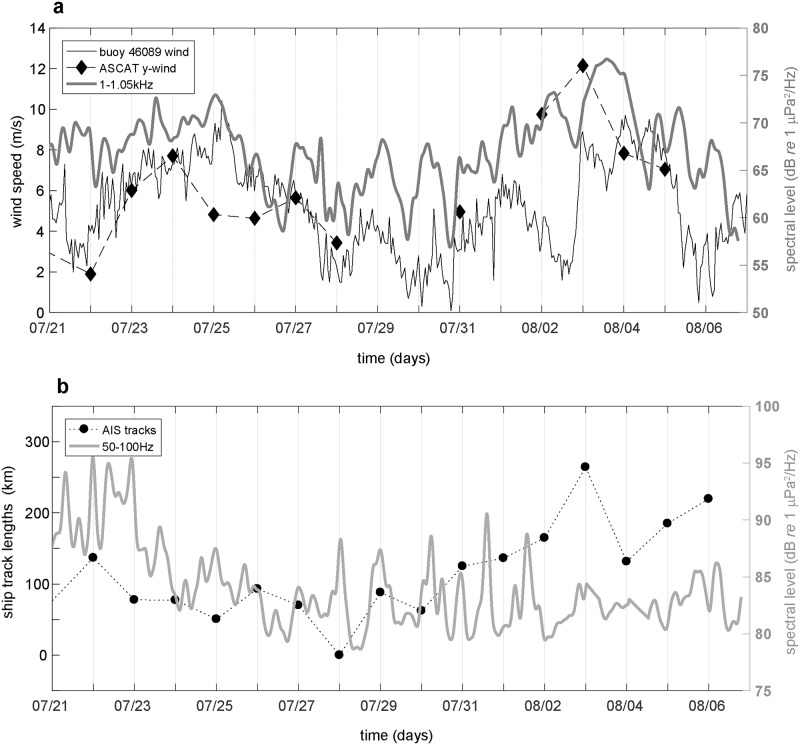
Noise level frequency band averages comparing ship and wind-dependent processes during the glider mission. **a)** Frequency band averaged (1–1.05 kHz) glider sound levels (thick line) plotted with wind speeds measured at buoy 46089 (thin line) and ASCAT (diamond-dash) satellite daily wind speed averages at 0.25° scale following the glider. **b)** Frequency band averaged (50–100 Hz) glider sound levels (thick line) and AIS ship track lengths within a 50 km radius of the daily median glider position (circle-dash). Averaged noise levels are smoothed with a 5 point median filter followed by a 6 hour low pass hann filter.

**Fig 6:**
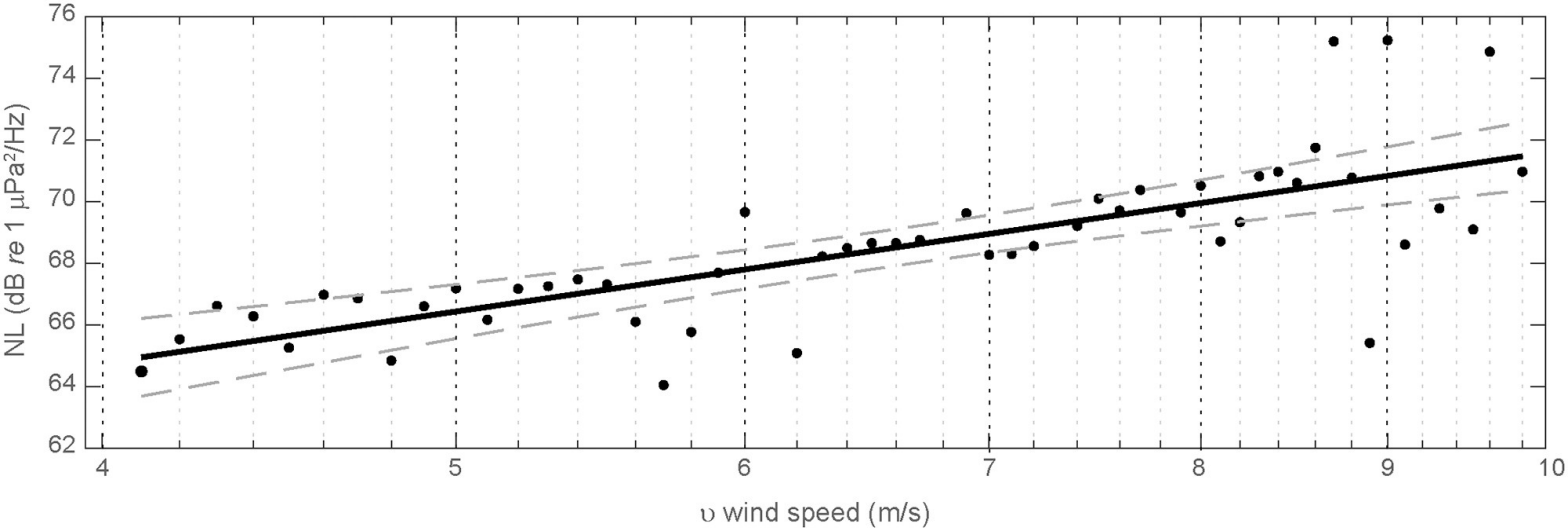
A linear least squares regression fit (R^2^ = 0.57, p << 0.05) of frequency band averaged (1–1.05 kHz) median noise levels recorded by the glider as a function of wind speeds measured at buoy 46089. Median noise levels are calculated from 10 cm/s bins and measurements from all depths are combined. 95% confidence intervals are shown as dashed lines.

Seasonal changes in surface wind speeds can greatly alter the underwater acoustic environment, substantially raising or lowering ocean sound levels within a region [22, 28, 29]. Along the continental shelf waters of the northeast Pacific, the summer upwelling season is driven by a strong northerly wind pattern often occurring with sustained speeds > 8 m/s and lasting several days [30]. During energetic conditions, wind speeds > 4–5 m/s mark the onset of surface wave breaking, the dominant process in wind-generated underwater ambient noise [31–34]. Piggott [35] showed for frequencies from a few hundred Hz up to 3 kHz, underwater noise levels scale with the power of the local wind speed of the form,

$${NL}_{f}(v)=B_{f}+20n_{f}\;\text{log}\;v$$ (1)

where the noise level (*NL*_*f*_) at a particular frequency or within a band is a function of the empirically derived parameters *B*_*f*_ and *n*_*f*_ and the logarithm of the wind speed *v*. Band averaged glider derived sound levels in the frequencies most influenced by surface winds (1–1.05 kHz) [22, 29] are in reasonable agreement with surface wind speeds despite the large separation distance from regional buoy and temporally coarse satellite measurements (Fig 5a). Median noise levels were calculated from 10 cm/s wind speed bins measured at buoy 46089 and applied to a linear least squares regression of Eq (1) to derive estimates for the model parameters *B*_(1−1.05*kHz*)_ = 54.4 and *n*_(1−1.05*kHz*)_ = 0.37. The relationship between wind speeds greater than 4 m/s associated with surface wave breaking and depth varying acoustic noise levels measured by the glider is supported with good model performance (R^2^ = 0.57, p<<0.05) including a majority of values falling within the 95% confidence intervals (Fig 6) resulting in a residual mean square error of 1.7 dB. As wind speeds reach higher velocities greater than 9 m/s, the data become more scattered suggesting other significant sources were present during these high wind speeds (e.g. vessels) or a transition to another mechanism of wind generated noise at higher speeds may become more important [13, 33]. Estimates of the scaling factor for the spectral slope (n_f_) of wind-dependent noise levels have typically been reported in the range of 0.5–1 for higher frequencies. The shallower spectral slope observed in this study (n_f_ = 0.37) is largely a consequence of including contributions from non-wind dependent acoustic sources (e.g. vessel noise) in the model fit.

**Fig 7:**
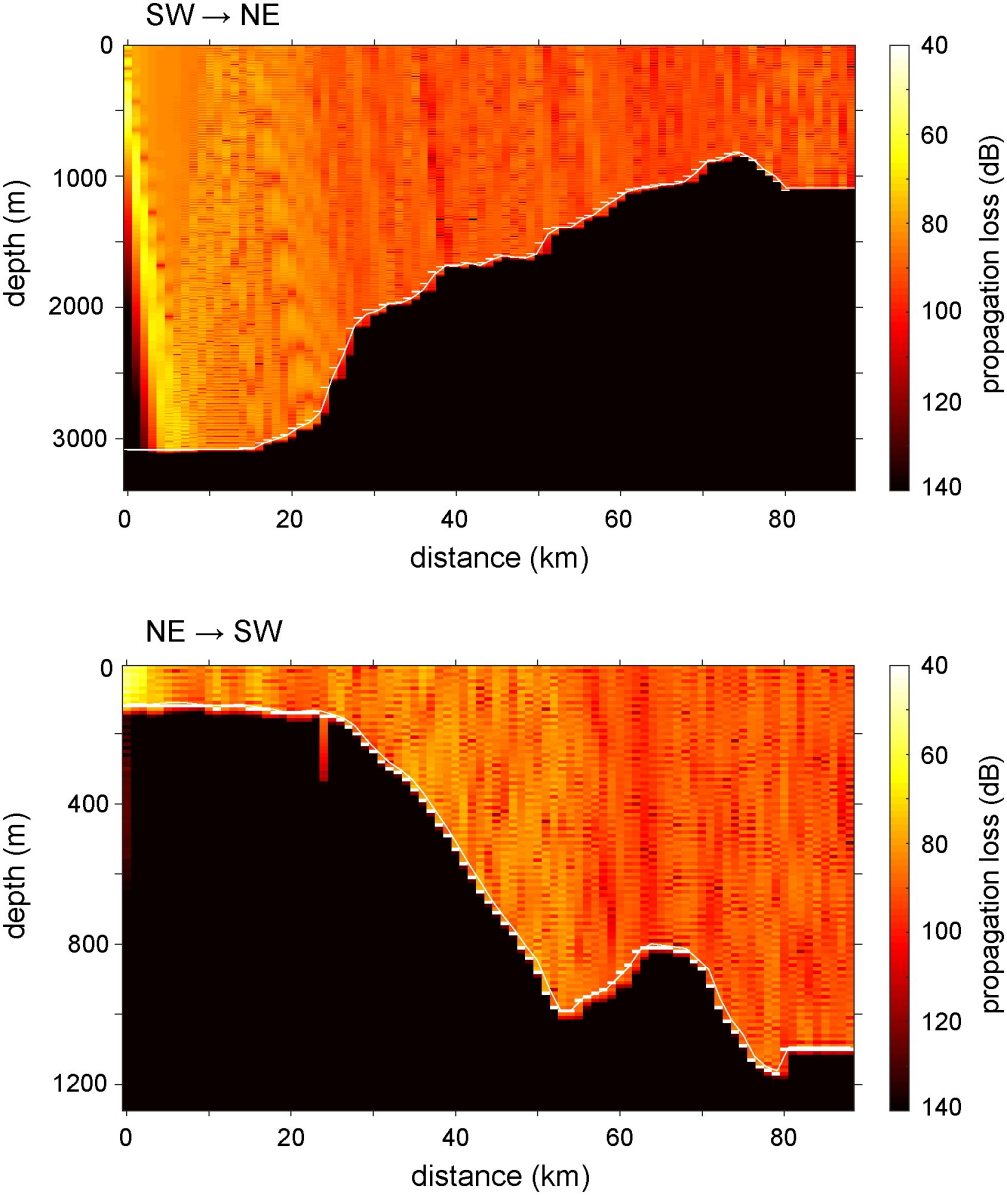
RAM propagation model output for ship noise generated from two vessel positions with respect to the glider (*f* = 100 Hz). **(upper)** Propagation loss from a range of 80 km SW of the glider traveling upslope from deeper water to the continental shelf break near 1000 m depth **(lower)** Energy loss from a range of 80 km to the NE of the glider propagating down the continental shelf toward the glider position. Sound source originated at 5 m depth.

As a result of the reduced effects of wind induced surface wave breaking on underwater low frequency (*f* < 100 Hz) [22] acoustic conditions, we examined averaged sound levels in the 50–100 Hz range that is heavily influenced by ship generated sounds [36, 37] and provided a reliable estimate for vessel related noise. Spectral energy in this “ship” noise frequency range remained high during the previously mentioned seismic airgun survey that occurred north of the southbound glider through July 23 (Fig 5b). The largest amount of variability in ship noise band acoustic levels was observed from July 28-Aug 2, and presumed to be from the nearby passage of vessels. Yet this higher ship band noise variability was not reflected by similar changes in the AIS derived daily ship track length parameter. In fact, as local ship track length increased to its peak during the glider mission on August 3, corresponding ship band noise levels appeared unaffected and returned to similar, steady values observed prior to the increase in variability observed near July 28. This discrepancy in the ship track length parameter and glider received noise levels was the result of propagation loss effects in acoustic energy radiated from vessels to the glider. Using glider measured sound speed profiles, local bathymetry and AIS vessel track information, 2D propagation loss estimates from the range dependent acoustic model (RAM) [38] showed that under the oceanographically stratified conditions found along the glider path, low frequency (100 Hz) acoustic energy attenuates by 80–90 dB within 10–20 km for surface generated (5m depth) sound sources (Fig 7). Therefore, as previously mentioned, since median noise levels at 100 Hz are measured around 85 dB re 1 μPa^2^/Hz (Fig 4) only the largest and loudest vessels (180 dB re 1 μPa) are capable of impacting glider received ambient noise levels outside of a 10–20 km radius. Noise radiated from smaller vessels is attenuated outside of this range and does not contribute significantly to ambient conditions. Furthermore, acoustic energy loss was greater for sources propagating eastward up the continental slope from deeper water toward the glider, than for vessel paths east of the glider on the continental shelf propagating downslope toward the glider (Fig 7). Additionally, a drawback of the ship band frequencies which are averaged over 50–100 Hz is that we were unable to clearly distinguish between the persistent contribution from distant, basin wide commercial shipping versus localized effects of vessel traffic transiting within 10’s of kilometers of the glider. Therefore, far-field shipping noise was assumed to be a stable feature of the low frequency acoustic spectra (*f* < 100 Hz) with superimposed changes in ship band energy levels resulting from noise radiated from local (< 50 km) vessel activity.

**Fig 8:**
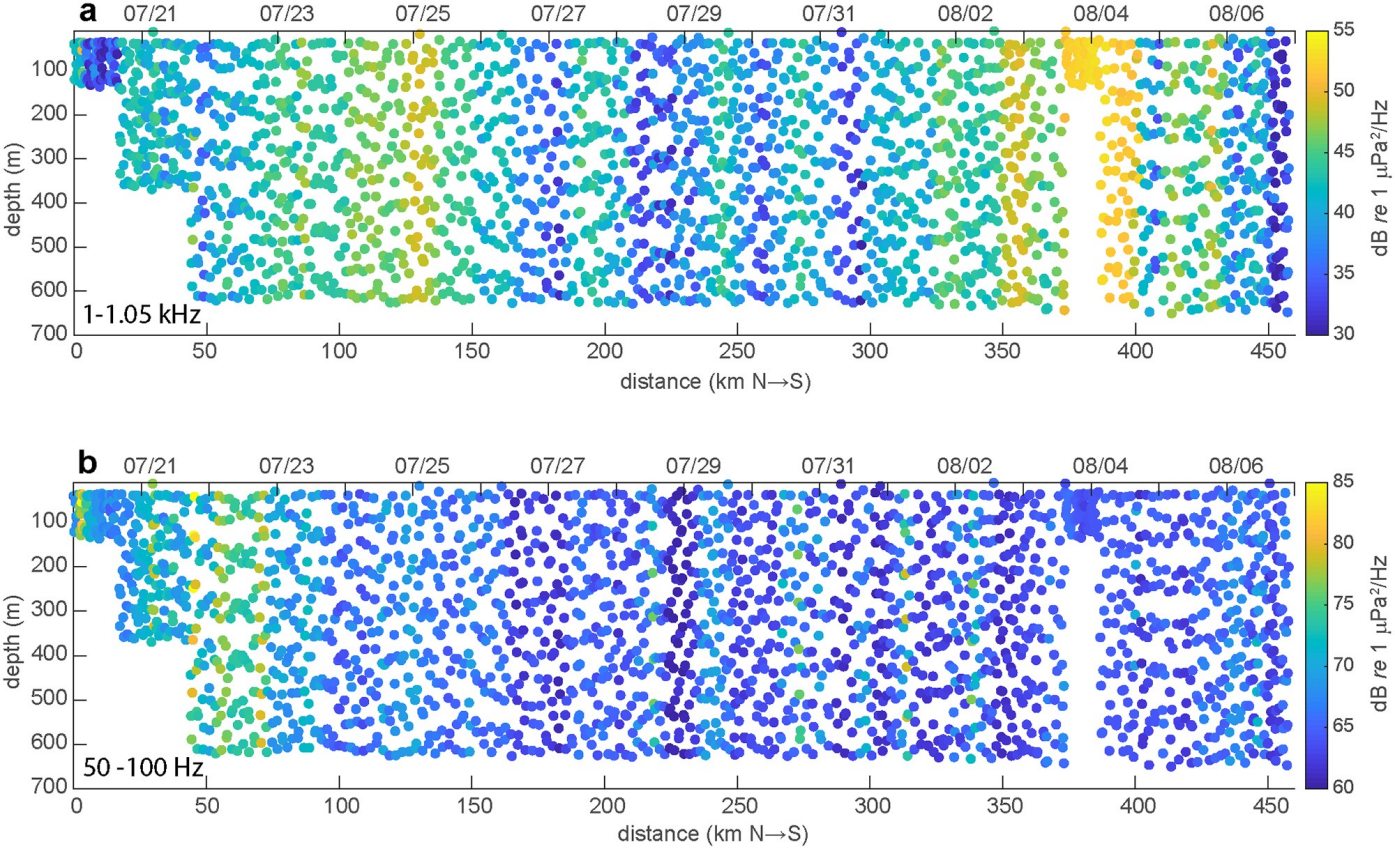
2-D scatter maps showing ambient conditions along the continental shelf break from the glider’s north to south progression. The **(a)** wind dependent frequency band and **(b)** ship noise band. Noise levels are calculated from 10 minute averages and the data during the mission along the glider track is shown on the upper x-axis. Note the change in energy scale between the upper and lower figures.

Scatter plots of wind and ship band sound levels (Fig 8) show the temporal and spatial patterns associated with surface generated processes and anthropogenic sources along the glider track. Elevated wind band conditions occurred intermittently, raising spectral levels on the order of 10 dB over periods of 1–3 days and showed little depth dependence. Meanwhile, ship band sound levels exhibited similar increases but occurred over much shorter intervals lasting only several hours instead of days, as expected with the passing of local ship traffic. Similar to wind dependent frequencies, ship band noise levels also exhibited limited variability throughout the 650 m glider depth profile.

**Fig 9:**
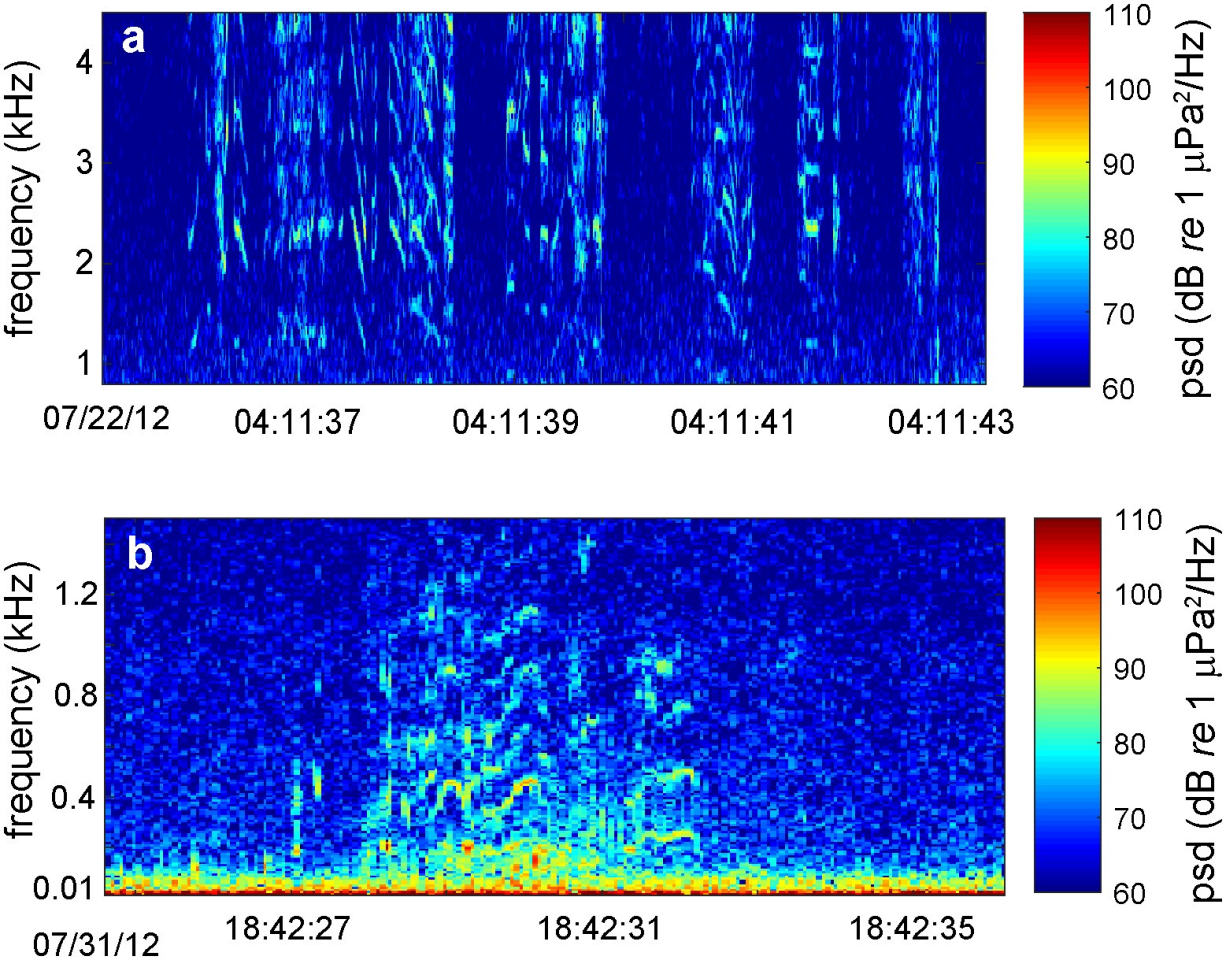
Spectrograms of bioacoustic signals observed along the glider mission. **(a)** 7 second long spectrogram of the low frequency component of commonly observed Pacific white-sided dolphin whistles (1–4.4 kHz); **(b)** an 11 second long spectrogram of low frequency (10–1500 Hz) humpback whale vocalizations recorded by the glider.

Biologically generated sounds including clicks, whistles and low frequency moans were observed at irregular intervals throughout the glider mission. The lower frequency component of Pacific white-sided dolphin (*Lagenorhynchus obliquidens*) whistles [39, 40] were the most commonly recorded bioacoustic signal with energy found mostly above 2 kHz (Fig 9a). A total of 11 vocal encounters with whistling dolphins occurred over the glider mission transect (Fig 1) with event durations lasting from between 5 minutes up to 3 hours. Often during the longer duration encounters, click trains were also observed indicative of foraging behavior. Curiously, the largest gap in time without a dolphin vocal encounter occurred between 45°- 44°N latitude from July 28–31, 2012 when the surface wind generated noise levels that overlap the dolphin frequencies were at a relative minimum and therefore acoustic detection probability was optimal, indicating the species had moved away from the area or was not vocally active. The second most commonly observed marine mammal signals were low frequency calls from humpback whales (*Megaptera novaeangliae*) (Fig 9b). These calls were mostly limited to frequencies less than 1 kHz [41] and found in 3 distinct areas separated by at least 100 km along the glider shelf transect (Fig 1). Vocal encounters lasted between 10 minutes to 1 hour with the longest event occurring near 44°N on July 31 a few hours prior to a dolphin vocal encounter after a large gap in bioacoustics signals. A possible reason for this return in vocal activity and species to the area could be related to the observed change in oceanographic conditions near July 31 at 44°N where the stratified layer begins to deepen after a wind relaxation (Figs 2 & 5a) and conditions become more favorable in the area for prey.

### Comparison with long-term acoustic measurements

**Fig 10:**
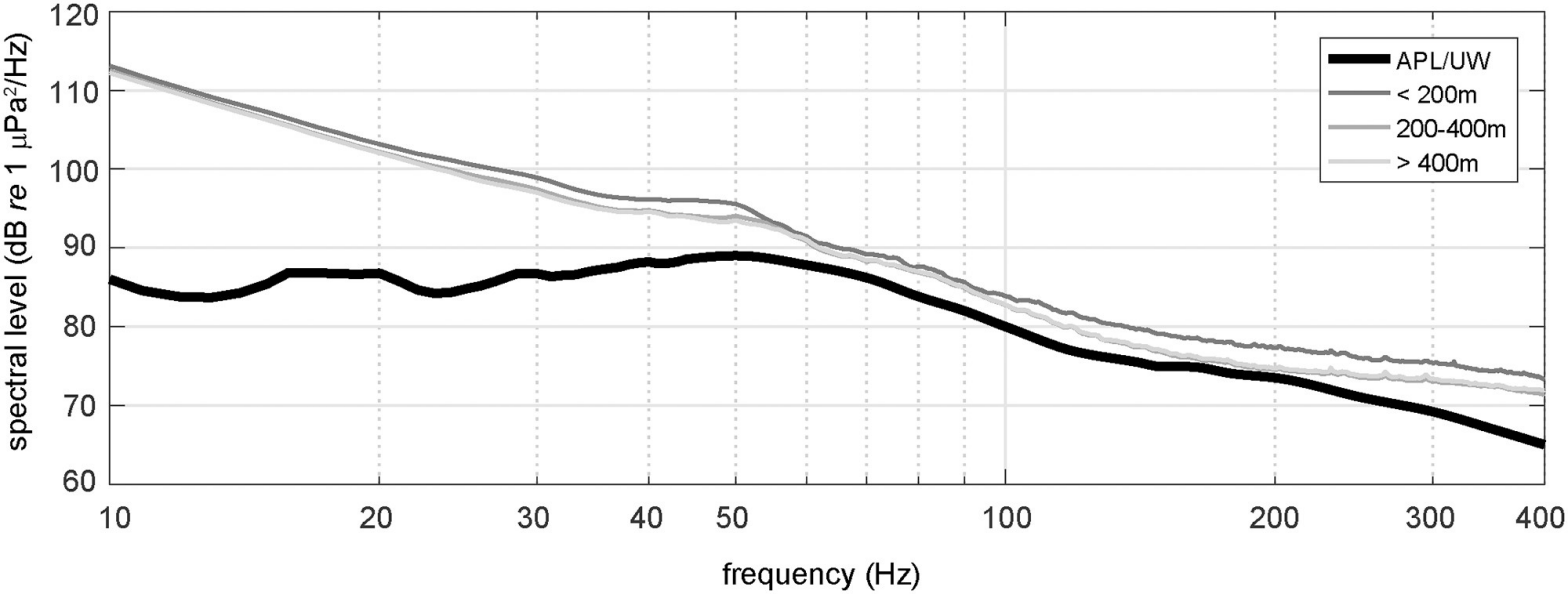
A comparison of median (50^th^ percentile) glider and fixed station spectral levels. Glider measurements from 3 depth intervals during the 2012 mission plotted against median spectra from a fixed, seafloor hydrophone system recorded from 1994–2007 near the same area in the northeast Pacific [18]. Values for APL/UW spectra taken from Andrew *et al*.’s, Fig 4 System *h*.

Long-term continuous measurements from moored hydrophones serve to reduce the effects of transient or extreme values in the acoustic characterization of a site. In comparison, averaging shorter-term, spatially distributed acoustic recordings over a larger regional area accessed by an ocean glider can reduce some of the inherent local variability in measured sound levels at low frequencies. Median spectral levels of the cumulative distribution (50^th^ percentile) from over 12 years of data (1994–2007) at a deepwater, fixed hydrophone array off the Oregon coast [18] provide a temporally robust baseline for ambient noise level comparisons with the short-term, yet spatially distributed median levels from glider recordings in the area during 2012. Glider derived median spectra (50%) from the shallow (10–200 m), mid (200–400 m) and deep (400–650 m) water cumulative distributions of Fig 4 are slightly higher than Andrew *et al*.’s 12 year median values for frequencies between 50–200 Hz (Fig 10). As previously mentioned, the significant rise in spectral slope and large discrepancy between glider measurements and Andrew *et al*.’s levels for frequencies below 50 Hz is attributed to flow noise contamination. Glider spectral slopes from 50–130 Hz match the fixed array, with differences in energy on the order of 2–5 dB *re* 1 μPa^2^/Hz, with the deeper glider recordings most consistent with fixed array values. The glider spectral levels begin to diverge from the long-term moored array for frequencies above 200 Hz where effects from winds and local vessel noise may have more influence on the shorter duration of the glider recordings.

## Discussion

While “snapshot” recordings from ocean gliders produce the most current measure of local or regional sound levels, decadal scale averages from fixed hydrophone arrays supply the background or context for variability in ambient noise levels through a range of acoustic conditions at a particular location. Only through repeated seasonal and multi-year visits of an acoustic glider to an area lacking fixed hydrophones could similar characterizations of noise variability could be achieved. Even so, stratification in the ambient noise field from the structure of the sound speed profile may result in significant differences in received levels between a hydrophone moored at depth and the depth varying glider sensor. Nevertheless, the side by side comparison of Andrew *et al*.’s long-term sound levels with glider derived measurements in this study are encouraging for the utilization of ocean gliders as an underwater noise measurement platform. Despite median spectra being consistently higher by a few dB in the 10 Hz– 400 Hz frequency range, the glider noise levels remain well within the 90^th^ percentile of cumulative distribution values from the 12 year fixed array measurements [18].

During the glider mission, significant contributions to ambient levels were recorded from seismic airguns, distant and local vessel radiated noise and wind generated surface processes. At times, complicated interactions between these sources resulted in wideband, elevated noise levels that were difficult to untangle. To best describe overall changes in ambient noise conditions, we did not restrict our analysis to discrete periods of ship or wind dependent noise in order to constrain the contributions from these sources [13, 33]. Instead, we incorporated all sources and focused on frequency bands influenced by particular processes. For example, sound levels in the 50–100 Hz frequency band were used as an indicator for near and far-field shipping activity, where contributions related to surface wind speeds were limited. Nevertheless, near the beginning of the glider mission this low frequency band was dominated by seismic airgun activity (Figs 5b and 8b). The frequency band 1–1.05 kHz used in our analysis to describe the influence of wind generated sound at the sea surface unfortunately also carries significant amount of local vessel noise (Fig 3c). Interestingly, biological signals from marine mammals common to the area did not have a significant impact on the distribution of spectral levels observed during the 18 day mission. Despite 11 vocal encounters with Pacific white-sided dolphins and 3 with humpback whales spread over the mission duration, the calling events were not persistent or high enough amplitude to sufficiently influence spectral levels over the time periods in this study. Similarly, the highly seasonal and consistent blue and fin whale vocalizations previously observed in this area of the northeast Pacific peak in activity during the fall months after the glider had transited the area [42, 43].

The ambient noise in the 50–100 Hz frequency band was not well correlated with the total daily ship track length metric from AIS data owed largely to significant attenuation loss along the propagation paths. RAM propagation loss model results indicated that smaller vessels transiting 10–20 km outside of the glider position had little to no effect on low frequency ambient noise levels, and that only the largest vessels with source levels near 180 dB re 1 μPa inside the 50 km buffer range could impact acoustic conditions near the glider. Additionally, the orientation of the vessel noise source east or west of the glider track along the continental shelf break had an impact on propagation loss where acoustic energy traveling upslope attenuated more rapidly and to a greater degree than energy loss downslope. The 50 km buffer for vessel track length incorporated larger vessel radiated noise, but introduced bias from smaller vessels that did not generate high enough amplitude sounds to be influential outside of the first 10–20 km from the source. Additionally, gaps in AIS transmissions can occur from poor quality equipment on the vessels or receiver station and can even be intentional on fishing vessels that disable their AIS systems to maintain privacy on fishing grounds [44] which could have also created inconsistencies between noise levels and total vessel track length.

Estimates of the relationship between surface winds and glider noise levels in the 1–1.05 kHz frequency band were challenged by the long distances between the glider and buoy 46089. Despite this physical separation, wind generated sounds were shown to be a consistent and predictable feature within the ambient spectra of the region, fluctuating as a function of the regionally representative proxy of measured wind speed at buoy 46089. Future glider missions targeting under water acoustic measures of surface wind speed magnitudes should focus on periods devoid of other sources (e.g. vessels) to further constrain this relationship. Furthermore, the transition from wind driven noise processes observed between 4–10 m/s to a second regime with wind speeds 10–20 m/s involving insulating bubble clouds and other high wind generated phenomenon near the surface need to be accounted for in order to properly describe the full range of influence on wind-dependent sound levels [13].

## Conclusions

Results from this study and another in the Mediterranean Sea [13], have shown the value of glider derived sound level measurements as a tool for climatic studies of ocean-atmosphere interaction using subsurface acoustic noise levels to predict sea surface wind speed magnitudes in remote ocean areas. Glider derived wind speed estimates from underwater acoustic measurements have the distinct advantage that they can be made far from destructive surface processes experienced by buoys. Therefore, in remote ocean areas, acoustic ocean gliders may be a robust and long-lived alternative to buoy measurements of surface winds requiring less maintenance and cost over time.

In addition to sea surface wind speed estimates, we’ve also shown that glider derived acoustic measurements are effective for characterizing regional-scale variability of ocean sound levelsin the northeast Pacific. Ocean gliders provide capacity for filling knowledge gaps on acoustic conditions where information is limited. Repeated glider surveys within a region of concern for acoustic habitat degradation can provide important real-time measurements of changing noise levels resulting from anthropogenic activities. Considering alternate mobile platforms capable of noise level assessments over large, remote ocean areas, ocean gliders provide significant advantages. For instance, the Wave Glider is a highly efficient, far ranging unmanned surface vehicle (USV) with towed hydrophone system capabilities. Nevertheless, the surface limited vehicle tows hydrophones at fixed, shallow depth (~10m)[45], where recordings are inherently contaminated from physical processes (waves, wind) and motion of the sensor. To enable accurate low frequency recordings and avoid flow-noise contamination from surface processes (wave orbital motions), the hydrophone sensors need to be decoupled from the vertical and horizontal accelerations of the USV. This is not required by the deep diving ocean gliders, as they move out of the active wave zone near the surface and whose relative slower motion reduces flow-noise in comparison to faster moving surface vehicles. Utilized as an alternative or supplement to long-term moored hydrophone arrays targeting temporal changes in particular marine environments [46], glider derived measurements offer enhanced spatial perspectives that are critical for informed management and regulatory decisions for acoustic habitat protection [47].

## Supporting information

S1 Appendix A method for the identification, removal and smoothing of glider rudder self-noise. (DOCX)

S1 Fig An example of the removal and smoothing algorithm for glider rudder noise contamination in the acoustic time series. Upper panel shows the raw acoustic waveform with rudder generated noise in black compared with the filtered and smoothed time series in gray. The lower panel shows the power spectra before and after the algorithm is applied, illustrating the significant reduction in rudder-generated self-noise contamination.
(TIF)

## References

[1] Wood SL, Mierzwa CE. State of Technology in Autonomous Underwater Gliders. Marine Technology Society Journal. 2013;47(5):84–96.

[2] Rudnick DL, Davis RE, Eriksen CC, Fratantoni DM, Perry MJ. Underwater gliders for ocean research. Marine Technology Society Journal. 2004;38(2):73–84.

[3] Davis RE, Eriksen CC, Jones CP. Autonomous buoyancy-driven underwater gliders. Taylor and Francis, London; 2002 p. 37–58.

[4] Schofield O, Kohut J, Aragon D, Creed L, Graver J, Haldeman C, et al Slocum gliders: Robust and ready. Journal of Field Robotics. 2007;24(6):473–85.

[5] Gebbie J, Siderius M, Allen JS. Aspect-dependent radiated noise analysis of an underway autonomous underwater vehicle. Journal of the Acoustical Society of America. 2012;132(5):El351–El7. 10.1121/1.4754419 23145694

[6] Baumgartner MF, Fratantoni DM. Diel periodicity in both sei whale vocalization rates and the vertical migration of their copepod prey observed from ocean gliders. Limnology and Oceanography. 2008;53(5part2):2197–209.

[7] Baumgartner MF, Fratantoni DM, Hurst TP, Brown MW, Cole TVN, Van Parijs SM, et al Real-time reporting of baleen whale passive acoustic detections from ocean gliders. The Journal of the Acoustical Society of America. 2013;134(3):1814–23. 10.1121/1.4816406 23967915

[8] Moore SE, Howe BM, Stafford KM, Boyd ML. Including whale call detection in standard ocean measurements: Application of acoustic Seagliders. Marine Technology Society Journal. 2007;41(4):53–7.

[9] Klinck H, Mellinger DK, Klinck K, Bogue NM, Luby JC, Jump WA, et al Near-real-time acoustic monitoring of beaked whales and other cetaceans using a Seaglider^™^. PloS one. 2012;7(5):e36128 10.1371/journal.pone.0036128 22629309 PMC3356361

[10] Küsel ET, Munoz T, Siderius M, Mellinger DK, Heimlich S. Marine mammal tracks from two-hydrophone acoustic recordings made with a glider. Ocean Science. 2017;13(2):273.

[11] Wall CC, Lembke C, Mann DA. Shelf-scale mapping of sound production by fishes in the eastern Gulf of Mexico, using autonomous glider technology. Marine Ecology Progress Series. 2012;449:55–64.

[12] Matsumoto H, Haxel JH, Dziak RP, Bohnenstiehl DR, Embley RW. Mapping the sound field of an erupting submarine volcano using an acoustic glider. The Journal of the Acoustical Society of America. 2011;129(3):EL94–EL9. 10.1121/1.3547720 21428474

[13] Cauchy P, Heywood KJ, Merchant ND, Queste BY, Testor P. Wind Speed Measured from Underwater Gliders Using Passive Acoustics. Journal of Atmospheric and Oceanic Technology. 2018;35(12):2305–21.

[14] Andrew RK, Howe BM, Mercer JA, Dzieciuch MA. Ocean ambient sound: comparing the 1960s with the 1990s for a receiver off the California coast. Acoustics Research Letters Online. 2002;3(2):65–70.

[15] McDonald MA, Hildebrand JA, Wiggins SM. Increases in deep ocean anibient noise in the northeast pacific west of San Nicolas Island, California. Journal of the Acoustical Society of America. 2006;120(2):711–8. 10.1121/1.2216565 16938959

[16] Curtis KR, Howe BM, Mercer JA. Low-frequency ambient sound in the North Pacific: Long time series observations. Journal of the Acoustical Society of America. 1999;106(6):3189–200.

[17] Chapman NR, Price A. Low frequency deep ocean ambient noise trend in the Northeast Pacific Ocean. The Journal of the Acoustical Society of America. 2011;129(5):EL161–EL5. 10.1121/1.3567084 21568369

[18] Andrew RK, Howe BM, Mercer JA. Long-time trends in ship traffic noise for four sites off the North American West Coast. Journal of the Acoustical Society of America. 2011;129(2):642–51. 10.1121/1.3518770 21361423

[19] Oliver MJ, Breece MW, Haulsee DE, Cimino MA, Kohut J, Aragon D, et al Factors affecting detection efficiency of mobile telemetry Slocum gliders. Animal Biotelemetry. 2017;5(1):14.

[20] Mellinger DK. Ishmael 1.0 user’s guide. NOAA Technical Memorandum OAR PMEL. 2001;120(26):98115–6349.

[21] Halpern D. Summertime surface diurnal period winds measured over an upwelling region near the Oregon coast. Journal of Geophysical Research. 1974;79(15):2223–30.

[22] Wenz GM. Acoustic ambient noise in the ocean: spectra and sources. The Journal of the Acoustical Society of America. 1962;34:1936.

[23] Hatch L, Clark C, Merrick R, Van Parijs S, Ponirakis D, Schwehr K, et al Characterizing the Relative Contributions of Large Vessels to Total Ocean Noise Fields: A Case Study Using the Gerry E. Studds Stellwagen Bank National Marine Sanctuary. Environ Manage. 2008;42(5):735–52. 10.1007/s00267-008-9169-4 18626686

[24] National Science Foundation (NSF) NOaAAN, Office of Naval Research (ONR), Schmidt Ocean Institute (SOI). Cascadia Open Access Seismic Transects (COAST) rvdataus. 2019;cruise MGL1212, R/V Marcus G. Langseth.

[25] Merchant ND, Barton TR, Thompson PM, Pirotta E, Dakin DT, Dorocicz J. Spectral probability density as a tool for ambient noise analysis. The Journal of the Acoustical Society of America. 2013;133(4):EL262–EL7. 10.1121/1.4794934 23556689

[26] Ross D. Mechanics of underwater sound. New York: Pergamon Press; 1976.

[27] Ross D. Ship sources of ambient noise. IEEE Journal of Oceanic Engineering. 2005;30(2):257–61.

[28] Knudsen VO, Alford R, Emling J. Underwater ambient noise. J Mar Res. 1948;7(3):410–29.

[29] Urick RJ. Principles of underwater sound, 1983. McGraw-Hill; 1983.

[30] Halpern D. Variations in the density field during coastal upwelling. Tethys. 1974;6(1–2):363–74.

[31] Carey WM, Browning D. Low frequency ocean ambient noise: measurements and theory Sea Surface Sound: Springer; 1988 p. 361–76.

[32] Kewley DJ, Browning DG, Carey WM. Low-frequency wind-generated ambient noise source levels. The Journal of the Acoustical Society of America. 1990;88(4):1894–902.

[33] Chapman NR, Cornish JW. Wind dependence of deep ocean ambient noise at low frequencies. The Journal of the Acoustical Society of America. 1993;93(2):782–9.

[34] Ma BB, Nystuen JA, Lien RC. Prediction of underwater sound levels from rain and wind. Journal of the Acoustical Society of America. 2005;117(6):3555–65. 10.1121/1.1910283 16018459

[35] Piggott C. Ambient sea noise at low frequencies in shallow water of the Scotian Shelf. The Journal of the Acoustical Society of America. 1964;36(11):2152–63.

[36] Sirovic A, Wiggins SM, Oleson EM. Ocean noise in the tropical and subtropical Pacific Ocean. Journal of the Acoustical Society of America. 2013;134(4):2681–9. 10.1121/1.4820884 24116406

[37] McKenna MF, Ross D, Wiggins SM, Hildebrand JA. Underwater radiated noise from modern commercial ships. Journal of the Acoustical Society of America. 2012;131(1):92–103. 10.1121/1.3664100 22280574

[38] Collins MD. New and improved parabolic equation models. The Journal of the Acoustical Society of America. 1998;104(3):1808-. 10.1121/1.423601

[39] Au WWL. The sonar of dolphins: Springer; 1993.

[40] Wiggins SM, Frasier KE, Elizabeth Henderson E, Hildebrand JA. Tracking dolphin whistles using an autonomous acoustic recorder array. The Journal of the Acoustical Society of America. 2013;133(6):3813–8. 10.1121/1.4802645 23742335

[41] Norris TF, Mc Donald M, Barlow J. Acoustic detections of singing humpback whales (Megaptera novaeangliae) in the eastern North Pacific during their northbound migration. The Journal of the Acoustical Society of America. 1999;106(1):506–14. 10.1121/1.427071 10420640

[42] Stafford KM, NieukIrk SL, Fox CG. Geographic and seasonal variation of blue whale calls in the North Pacific. Journal of Cetacean Research and Management. 2001;3(1):65–76.

[43] Moore SE, Stafford KM, Dahlheim ME, Fox CG, Braham HW, Polovina JJ, et al Seasonal variation in reception of fin whale calls at five geographic areas in the North Pacific. Marine Mammal Science. 2006;14(3):617–27.

[44] Ford JH, Peel D, Kroodsma D, Hardesty BD, Rosebrock U, Wilcox C. Detecting suspicious activities at sea based on anomalies in Automatic Identification Systems transmissions. Plos One. 2018;13(8). 10.1371/journal.pone.0201640 PMC6084947 30091985

[45] Wiggins S, Manley J, Brager E, Woolhiser B, editors. Monitoring marine mammal acoustics using Wave Glider. OCEANS 2010 MTS/IEEE SEATTLE; 2010 20–23 Sept. 2010.

[46] Haver SM, Gedamke J, Hatch LT, Dziak RP, Van Parijs S, McKenna MF, et al Monitoring long-term soundscape trends in US Waters: The NOAA/NPS Ocean Noise Reference Station Network. Marine Policy. 2018;90:6–13.

[47] Hatch LT, Wahle CM, Gedamke J, Harrison J, Laws B, Moore SE, et al Can you hear me here? Managing acoustic habitat in US waters. Endangered Species Research. 2016;30:171–86.

