# Supplementary material for: Ocean sound levels in the northeast Pacific recorded from an autonomous underwater glider
Source: PLoS One. 2019 Nov 20;14(11):e0225325. doi: 10.1371/journal.pone.0225325 (PMC6867629; doi:10.1371/journal.pone.0225325)
Supplement: S1 Appendix — (DOCX) [file pone.0225325.s001.docx]

**S1 Appendix**

Due to its short duration, rudder self-noise could be identified, filtered and smoothed to maintain a more consistent acoustic time series in comparison with longer duration pump and battery noise which resulted in data loss. Rudder-generated, glider self-noise contamination was identified using a simple, frequency band limited (10 Hz < *f* < 1000 Hz), energy summation automated detection algorithm in the acoustic software Ishmael (20). Acoustic data was normalized using a 5 second spectrogram equalization window to remove long, continuous noises such as ships and weather. Acoustic energy levels within the frequency band from 10 Hz – 1000 Hz were summed at 0.1 second intervals triggering a detection event after crossing a unitless, empirical threshold of 0.25 above the mean normalized energy level of the detection function (20). The signal length detection window was set from 0.1-100 seconds in order to capture an extended range of glider system noise in addition to the rudder generated signals. When a detection event occurred, 1 second of data encompassing either side of the detection duration period was saved as a separate file and recorded in a log. Each of these automated detections was quality checked and visually annotated from time series and spectrogram displays to identify the origin of the signal yielding 33,441 long and short duration rudder noise events over the 18 day acoustic recording period.

A weighted smoothing algorithm was used to reduce contamination effects of the glider rudder self-noise. For each rudder noise event identified by the automated detection algorithm, the total number of data points in the signal, *N_d_*, was determined using the start time (*t_1_*) and end time (*t_2_*) from the detector (eq. S1) where *x(t)* was the acoustic pressure time series at time *t*.

$N_{d}=\left[ x\left( t_{1} \right),\ldots x\left( t_{2} \right) \right]$ (S1)

To ensure the capture of the complete duration of the rudder noise, ± 0.5 second buffer on either side of the automated detection boundaries (*t_1_*, *t_2_*) was added to derive the rudder noise signal *N_r_* of length *n* and temporal period *T_r_*.

$N_{r}=\left[ x\left( t_{1}-0.5 sec \right),\ldots x\left( t_{2}+0.5 sec \right) \right]$ (S2)

with the temporal range of *N_r_* defined as

$\left( t_{2}+0.5 sec \right)-\left( t_{1}-0.5 sec \right)=t_{2}-t_{1}+1 sec=T_{r}$ (S3)

Data periods *N_b_* and *N_a_* were defined as periods where *N_b_* was the range of *n* data points found before the beginning of *Nr*. Similarly, *N_a_* was the range of *n* data points occurring after the end of *N_r_*.

$N_{b}=[x(t_{1}-T_{r}-0.5sec),\ldots,x\left( t_{1}-0.5 \sec\right)]$ (S4)

$N_{a}=[x(t_{2}+0.5\sec),\ldots,x(t_{2}+T_{r}+0.5\sec)]$ (S5)

A weighted average of the acoustic energy levels in the data surrounding the rudder noise event (*N_a_, N_b_*) was substituted for the contamination period, effectively removing the rudder generated noise from the data while preserving the time series throughout the length of the deployment (eq. S6).

$N_{r}=\frac{\left[ N_{a}+N_{b} \right]}{1.25}$ (S6)

Comparison of the waveforms and power spectra from the raw and rudder noise corrected data show a significant reduction in contamination of ambient levels from the rudder servo motor (S1 Fig) enabling a more accurate recording of the true sound levels by the glider PAM system.
