# Supplementary figures and images for: Ocean sound levels in the northeast Pacific recorded from an autonomous underwater glider
Source: PLoS One. 2019 Nov 20;14(11):e0225325. doi: 10.1371/journal.pone.0225325 (PMC6867629; doi:10.1371/journal.pone.0225325)

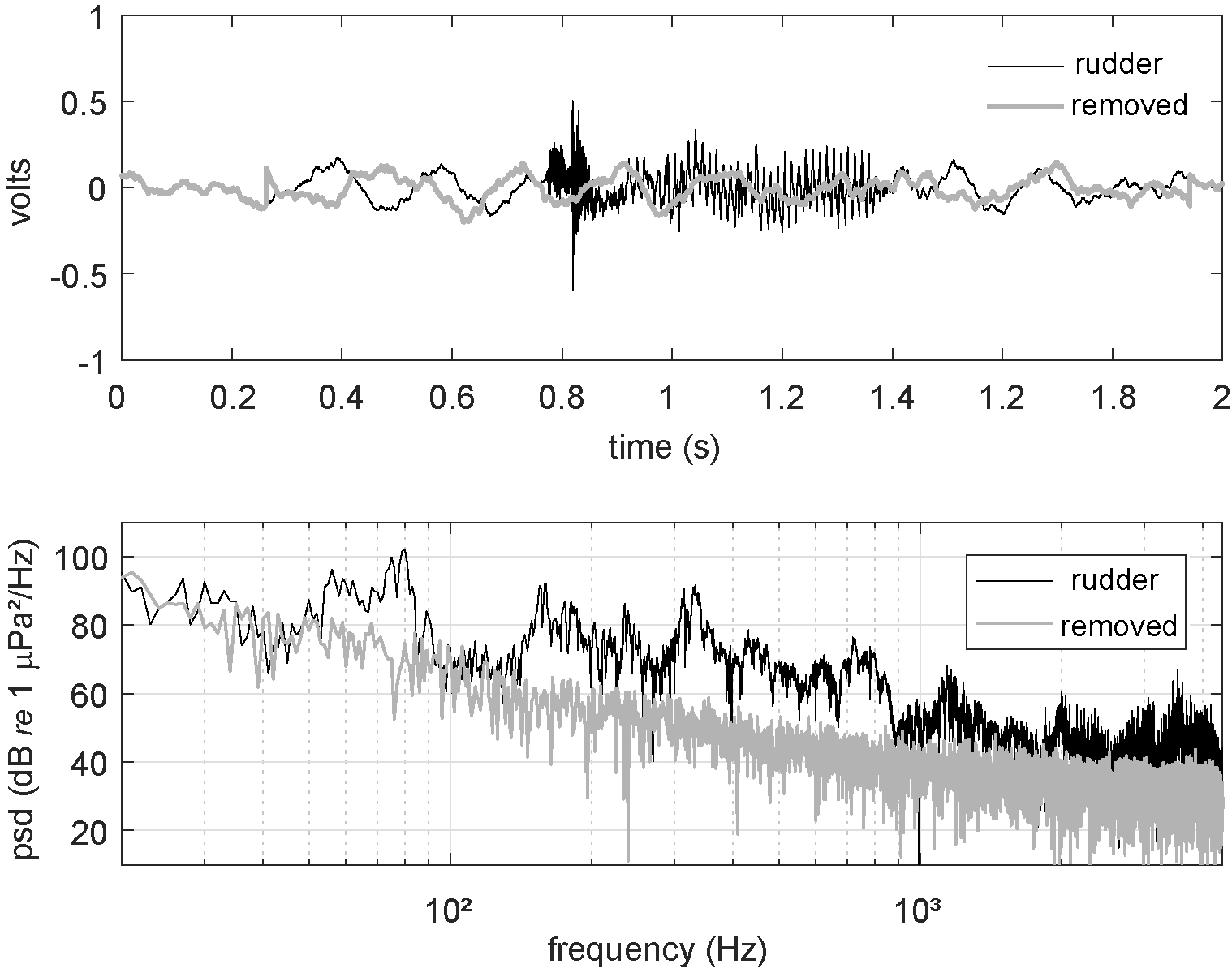

Supplement: S1 Fig — Upper panel shows the raw acoustic waveform with rudder generated noise in black compared with the filtered and smoothed time series in gray. The lower panel shows the power spectra before and after the algorithm is applied, illustrating the significant reduction in rudder-generated self-noise contamination. (TIF) [file pone.0225325.s002.tif]
